# An Unusual Form of a Trichobezoar Causing a Peculiar Case of Appendicitis: A Case of Rapunzel Syndrome

**DOI:** 10.7759/cureus.7554

**Published:** 2020-04-06

**Authors:** Gabriel Michael, Ehsaan Qureshi, Mohammad Miah, Najam Husain

**Affiliations:** 1 General Surgery, University Hospitals of Derby and Burton National Health Service (NHS) Foundation Trust, Burton on Trent, GBR; 2 General Surgery, University Hospitals of Derby and Burton National Health Service (NHS) Foundation Trust, Burton on Trent, GBR; 3 General and Colorectal Surgery, University Hospitals of Derby and Burton National Health Service (NHS) Foundation Trust, Burton on Trent, GBR

**Keywords:** appendicitis, rapunzel syndrome, surgery, trichotillomania, trichobezoar

## Abstract

Rapunzel syndrome is an extremely rare intestinal condition stemming from trichophagia (compulsive ingestion of hair). The syndrome usually causes intestinal obstruction but we report a unique case where it has caused appendicitis. We also reviewed the existing literature on Rapunzel syndrome.

## Introduction

Originally described in 1968, Rapunzel syndrome is a condition where the body of a trichobezoar is found within the proximal aspect of the gastrointestinal tract, with a tail that extends into the small intestine. It has been described to occur predominantly among females, adolescent patients, and occurs as a result of trichotillomania (compulsive hair plucking) and trichophagia (hair swallowing) [1-2]. Whilst trichotillomania is now a recognized diagnosis according to the ICD-10 criteria, patients may have associated psychiatric conditions such as anxiety disorders, obsessive compulsive disorder, depression, and anorexia nervosa.

Patients can present with a number of symptoms, ranging from a painless abdominal mass, early satiety, to chronic abdominal pain, weight loss, vomiting, and even obstruction or perforation. Complications of Rapunzel syndrome may include: intestinal obstruction, intussusception, gastric ulceration, cholestatic jaundice, or acute pancreatitis [3-5]. A suspicion of trichophagia, as suggested by apparent alopecia, in the presence of clinical signs may lead to a consideration of Rapunzel syndrome as a diagnosis. However, alopecia may not always be present, and therefore direct questioning when eliciting a history may be required. In our case there was no alopecia, and clinical signs were more suggestive of appendicitis.

The diagnosis of Rapunzel syndrome may be further supported by imaging modalities such as an abdominal X-ray, or a CT scan, but an endoscopy would confirm the diagnosis as the trichobezoar would be visualized.

Treatment is through the removal of the bezoar. In most cases, laparotomy is necessary as the bezoar is too large to be removed endoscopically or laparoscopically. In this case, along with the patient presentation there was no indication or signs exhibited by the patient that trichotillomania was taking place. 

## Case presentation

A 37-year-old Caucasian women presented with a 12-18 h history of initial peri-umbilical pain that soon migrated towards the right iliac fossa (RIF). This pain progressively worsened over the initial 12-18 h period. No lower urinary tract symptoms, abnormalities in opening bowel, or loss of appetite was noted. The patient was not nauseous and did not experience any episodes of vomiting. The only past medical history to be noted at this time was the previous miscarriage that the patient had eight months prior to this admission.

Local peritonism in the RIF was elicited upon abdominal examination with no other abnormalities to note. The patient was afebrile and no other signs were elicited on general examination. With the initial blood results available (as seen in Table 1) the surgical plan was to admit the patient and prepare her for a laparoscopic appendicectomy. At this stage STAT doses of co-amoxiclav and metronidazole antibiotics were given to the patient for infection control purposes.

**Table 1:** Laboratory results as on 24/05/2019 taken at 12:24 PM. WBC, white blood cell; GFR, glomerular filtration rate; ALT, alanine transaminase; ALP, alkaline phosphatase; CRP, C-reactive protein

Lab test	Patient results	Physiological range
WBC	14.6	4.0-10.0 (10*9/L)
Platelet count	169	150-410 (10*9/L)
Neutrophil count	12.3	2.0-7.0 (10*9/L)
Hemoglobin	122	120-150 (g/L)
Mean corpuscular volume	78.7	83.0-101.0 (fL)
Sodium	138	133-146 (mmol/L)
Potassium	3.6	3.5-5.3 (mmol/L)
Urea	3.6	2.5-7.9 (mmol/L)
Estimated GFR (eGFR)	106.9	90-120 (mL/min)
Total bilirubin	18	0-21 (umol/L)
ALT	30	0-33 (U/L)
ALP	68	30-130 (U/L)
CRP	48	0-5 (mg/L)
Total amylase	14	28-100 (U/L)

Due to the theater workload, the patient’s procedure did not take place until 6 h and 31 min after being initially reviewed and admitted. During this time the patient deteriorated clinically; complaining of an increasing intensity in the level of pain, as well as spiking a temperature of 38.5 degrees Celsius. This warranted a repeat blood test (which did not show an increase in inflammatory markers), and prioritizing the patient for theater as well as an arterial blood gas (ABG); the results of which can be viewed below (Table 2).

**Table 2:** Patient's ABG results. ABG, arterial blood gas

	Patient results	Physiological range
pH	7.46	7.35-7.45
pCO2	4.2	4-6 (kPa)
pO2	33.0	10-13 (kPa)
Lactate	2.1	0.5-1.9 (mmol/L)
O2 saturation	98.9%	95%-100%
Base excess	-0.5	+/- 2 (mmol/L)

**Figure 1:**
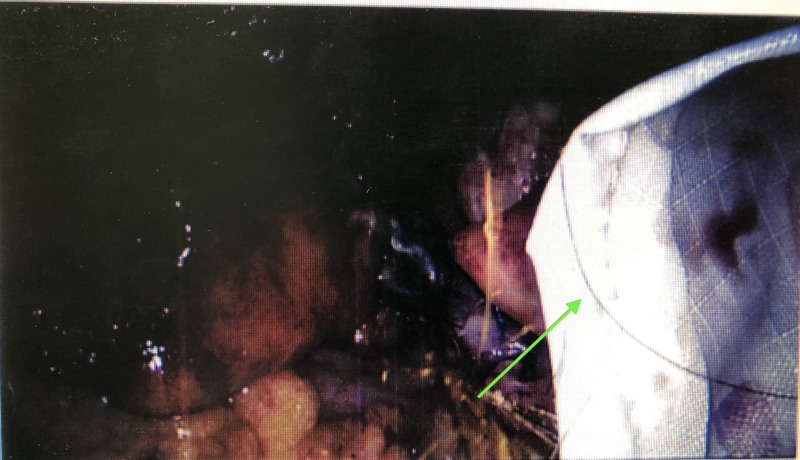
Initial strand of hair observed in BERT bag.

**Figure 2:**
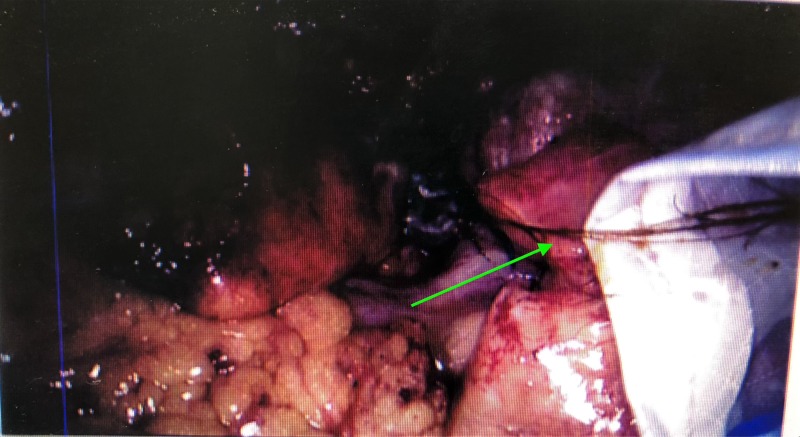
Further strands of hair being transferred into the BERT bag.

**Figure 3:**
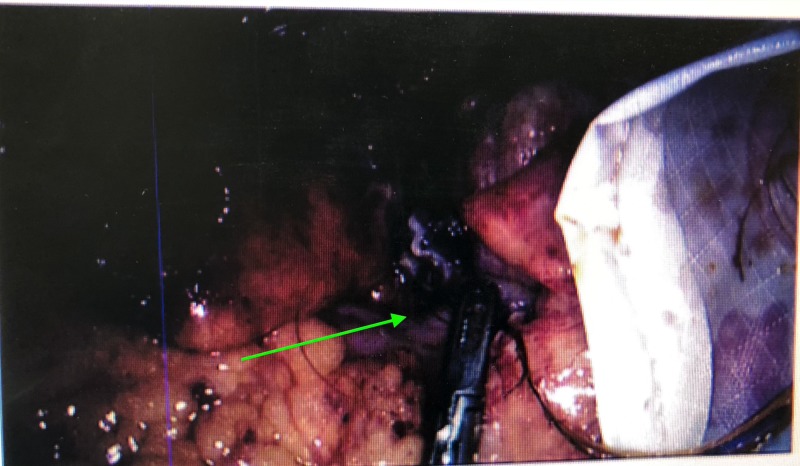
Grasper at the level of the appendix base demonstrating further hair.

**Figure 4:**
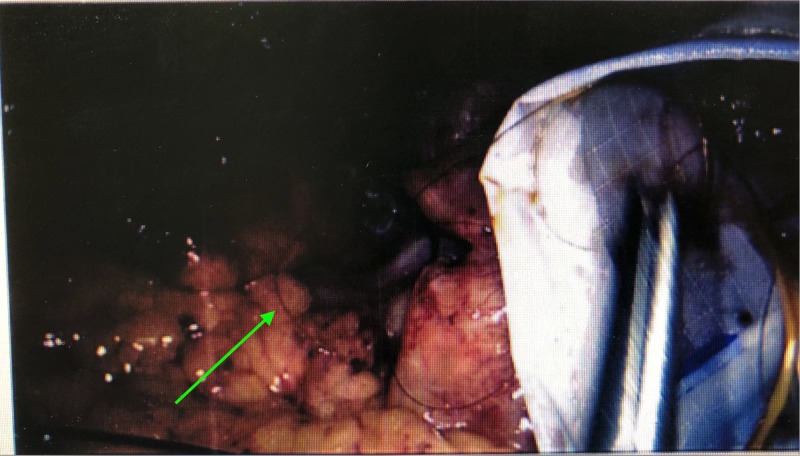
Further clearing of hair from peritoneal cavity.

**Figure 5:**
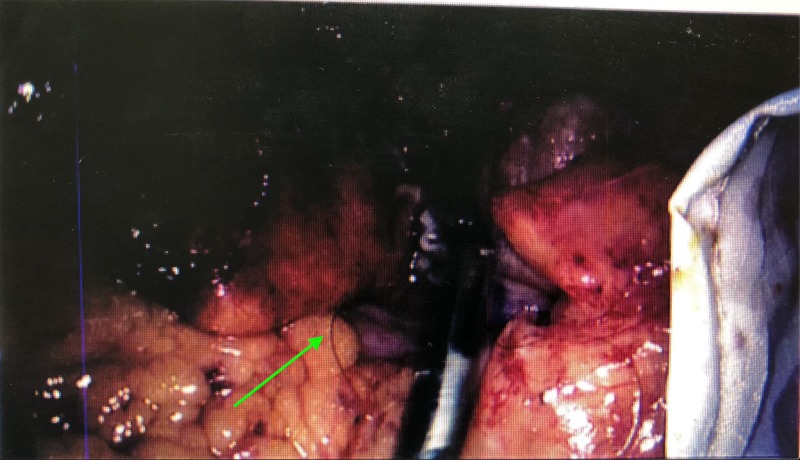
Further hair being removed from appendiceal base.

When the patient was taken to the theater, a standard approach was used for the laparoscopic procedure with an uneventful port insertion. The findings upon insertion of the laparoscopic camera into the abdomen were quite surprising to the surgical team. An inflammatory mass was visible at the RIF. The appendix was clearly gangrenous and perforated during its manipulation. At this point we must also note a hair follicle at the base of the appendix as well as a faecolith (as shown in Figures 1-5). Whilst all the visible hair was removed and all quadrants were washed a drain still had to be inserted into the pelvis [6-9].

Postoperatively the patient was admitted to the intensive treatment unit (ITU) for three days. During this time the patient was still septic and was being treated with IV antibiotics. During recovery in ITU the patient was approached and questions were asked with regard to the findings intra-operatively. At this point the patient did admit to voluntarily ingesting her own hair (a process known as trichophagia). Trichophagia is commonly accompanied with trichotillomania (pulling of one’s hair), although in this case the patient did not have the typical signs of such actions (friar tuck sign). It was also noted at this time that the surrounding family members were not aware of these actions and therefore oblivious to its consequences.

 After being stepped down from the ITU ward the patient recovered and was subsequently discharged after staying a further two days. No repeat blood tests were obtained at this point as the patient was clinically well. Their general practitioner (GP) was made aware of the admission, as well as advised to ensure that the patient seeks appropriate medical help for the ongoing conditions. This was quite an important step as merely maintaining on the current course would ensure re-admission with a worse and potentially fatal diagnosis.

## Discussion

Rapunzel syndrome was first described in literature in 1968 by the following characteristics:

- The body of a trichobezoar is ingested with a tail that can be found in the small intestines

- Small or large bowel obstruction

- Occurring in psychiatric patients

- Trichotillomania.

We must, however, note that not all the characteristics may be present or may be well hidden by the presenting individuals (such as the one in this case). The typical symptoms in the late stages include: chronic abdominal pain, stomach ulcers, perforation, stomach bleeding, intussusception, and obstruction. Associated psychiatric disorders that may coexist with trichotillomania and trichophagia are as follows: obsessive compulsive disorder, depression, anorexia nervosa, and depression.

 Whilst a trichobezoar can be diagnosed preoperatively, endoscopy is the usual method of diagnosis of the syndrome [10]. We can also note that CT imaging can be useful in determining the size, location, and extension of the trichobezoar. The treatment of these trichobezoars often consists of their removal. Although other noninvasive techniques such as medical treatment and enzymatic dissolution have been attempted, their success rates were actually quite low. Endoscopy has also failed in this case as often the trichobezoars are too large to be removed in such a manner. We note that in this case the patient did not undergo endoscopy as they were presenting with appendicitis, and at that time were treated as such. In some endoscopic cases the complications such as: pressure ulcers, esophagitis, and esophageal perforation were also observed. In this case we were fortunate to only require a laparoscopic technique for the removal of the small trichobezoar, as in the cases of larger more complex trichobezoars a laparotomy would be required. This would contribute towards a greater operative risk as well as a reduced cosmesis of the surgical incisions.

## Conclusions

 In this case psychiatric evaluation was performed postoperatively as there was nothing to suggest at the time when the patient had trichophagia. However, it would be best to have evaluated the patient prior to the procedure in order to ensure that the most appropriate medical treatment could be given along with the surgical intervention. Typically cognitive behavioral therapy should be considered in patients with trichophagia.
